# Is the Antitumor Property of *Trypanosoma cruzi* Infection Mediated by Its Calreticulin?

**DOI:** 10.3389/fimmu.2016.00268

**Published:** 2016-07-11

**Authors:** Galia Ramírez-Toloza, Paula Abello, Arturo Ferreira

**Affiliations:** ^1^Faculty of Veterinary Medicine and Livestock Sciences, University of Chile, Santiago, Chile; ^2^Program of Immunology, Faculty of Medicine, Institute of Biomedical Sciences (ICBM), University of Chile, Santiago, Chile

**Keywords:** calreticulin, *Trypanosoma cruzi*, trypomastigotes, complement system, C1q, cC1qR, tumor growth, immune response

## Abstract

Eight to 10 million people in 21 endemic countries are infected with *Trypanosoma cruzi*. However, only 30% of those infected develop symptoms of Chagas’ disease, a chronic, neglected tropical disease worldwide. Similar to other pathogens, *T. cruzi* has evolved to resist the host immune response. Studies, performed 80 years ago in the Soviet Union, proposed that *T. cruzi* infects tumor cells with similar capacity to that displayed for target tissues such as cardiac, aortic, or digestive. An antagonistic relationship between *T. cruzi* infection and cancer development was also proposed, but the molecular mechanisms involved have remained largely unknown. Probably, a variety of *T. cruzi* molecules is involved. This review focuses on how *T. cruzi* calreticulin (TcCRT), exteriorized from the endoplasmic reticulum, targets the first classical complement component C1 and negatively regulates the classical complement activation cascade, promoting parasite infectivity. We propose that this C1-dependent TcCRT-mediated virulence is critical to explain, at least an important part, of the parasite capacity to inhibit tumor development. We will discuss how TcCRT, by directly interacting with venous and arterial endothelial cells, inhibits angiogenesis and tumor growth. Thus, these TcCRT functions not only illustrate *T. cruzi* interactions with the host immune defensive strategies, but also illustrate a possible co-evolutionary adaptation to privilege a prolonged interaction with its host.

## Introduction

*Trypanosoma cruzi* (the protozoan agent of Chagas’ disease) cell infection is preceded by a variety of molecular interactions (1). Of relevance is the generation of a synapsis involving parasite endoplasmic reticulum (ER)-resident *T. cruzi* calreticulin (TcCRT) that, after translocation, interacts with complement component C1. C1 is then inactivated and recognized by cC1qR (a membrane form of mammalian CRT). The complement system, an important arm of innate and adaptive immune responses, is thus inhibited and parasite infectivity increased.

A significant decrease in experimental tumor growth is observed in experimental animals treated with recombinant TcCRT (rTcCRT) or infected with *T. cruzi*. A unifying molecular basis for these apparently unrelated phenomena is proposed herein. These molecular interactions do provide benefits for both the host and the parasite.

Through evolution, microbial agents have developed different mechanisms to resist the host immune response. In apparently unrelated strategies, some infectious agents elicit antitumor immune responses, leading to inhibition of cancer progression (2). Although these antitumor effects have been reported for several decades now, for a variety of infections, information on pathogen molecules involved is scarce (3).

Eight to 10 million people in 21 endemic countries are infected with *T. cruzi*. In about 30% of those infected, manifests, Chagas’ disease, a worldwide neglected tropical chronic illness (4, 5). The disease, originally endemic in Latin America, is now global, mainly because of migrations to USA, Canada, Europe, Oceania, and Asia (6), where transmission is mainly through blood transfusions, organ transplants, or congenital (7).

Eighty years ago, it was proposed that *T. cruzi* possesses an anticancer activity. Several *T. cruzi* strains displayed growth inhibitory effects over multiple transplanted or spontaneous tumors, in animal experimental models and humans (8, 9). This property was attributed to a “toxic substance” secreted by the parasite (10, 11). This “toxin” reduced pain, tumor growth, bleeding, and local inflammation in humans affected by a variety of tumors (12).

Chronically infected rats are more resistant to a carcinoma induced by 1,2-dimethylhidrazyne (9), and *T. cruzi* has a tropism for tumor cells, suggesting an antagonistic relationship between Chagas’ disease and cancer development (8). Elemental Darwinian reasoning allows us to propose that, if host survival is favored, chances for improved parasite persistence are evident.

Some authors have proposed that tumor and parasites compete for nutrients with consequent inhibition of tumor growth (13). However, this hypothesis is not entirely satisfactory since tumor growth is a multistep and complex process involving development of new blood vessels (angiogenesis) that provide the tumor with the necessary nutrients, oxygen, and means for waste removal (14). Other investigators have demonstrated, using a recombinant non-pathogenic *T. cruzi* clone as vector of a testis tumor antigen, the activation of T cell-mediated immunity. This specific cell immunity could delay tumor development in infected mice (15). In this work, it would have been important to define whether the non-pathogenic *T. cruzi* clone used translocates-externalizes its CRT. Non-infective epimastigotes are strongly impaired in their capacity to translocate this chaperone (16). Moreover, hemiallelic *TcCRT* KO, wild type, and transgenic parasites, respectively carrying one, two, and three *TcCRT* gene copies, express increased levels of the protein, *in vitro* resistance to human complement, and higher infectivity (16, 17).

Most likely, multiple parasite molecules and mechanisms are involved in the tumor resistance mediated by *T. cruzi* infection. Understanding these mechanisms may contribute to identify new therapeutic targets against cancer and Chagas’ disease.

**Figure 1:**
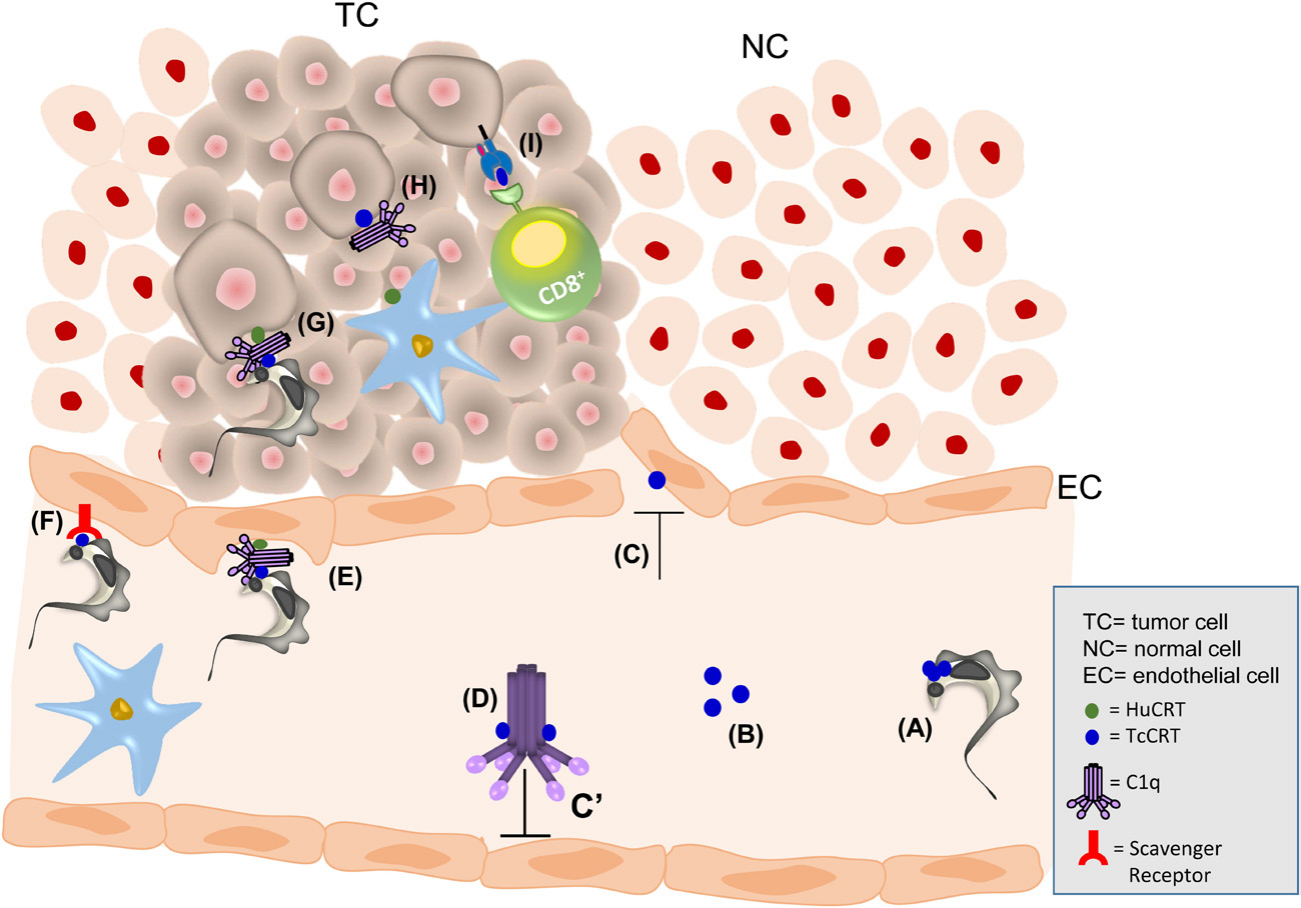
**The antitumor effect of *T. cruzi* infection may be explained by TcCRT**. TcCRT is exposed on the parasite surface (A) and secreted (B). TcCRT inhibits angiogenesis **(C)** and the activation of the classical pathway of the complement system through C1 inactivation **(D)**. TcCRT, present on the parasite surface, recruits C1. On the EC membrane, a trimolecular synapse is formed by HuCRT/C1q/TcCRT. This interaction increases the infectivity process **(E)**. TcCRT is also recognized by SRs on ECs, promoting infectivity **(F)**. The HuCRT/C1q/TcCRT interaction can also promote *T. cruzi* infectivity in TCs **(G)**. Moreover, TcCRT could mediate induction of an anamnestic antitumor immune response. Parasite could translocate TcCRT bound to the tumor cell with subsequent capture of host C1 **(H)**. This C1 will be recognized by HuCRT present on an antigen-presenting cell (APC), followed by internalization of this complex. Among many other possibilities, APCs will cross-process TcCRT, and specific peptides from this parasite protein will be loaded onto MHC I molecules. APCs will enter the regional lymph node and present these nTcCRT-specific peptides to cytotoxic T lymphocytes, thus leading to their activation. These CD8^+^ cytotoxic T lymphocytes will leave the lymph node and kill tumor cells that also present TcCRT-derived peptides **(I)**.

Our laboratory has been working for more than 20 years now with TcCRT, a multifunctional ER-resident protein that the parasite translocates to the external milieu (as depicted in Figures 1A,B). TcCRT is involved in a multiplicity of host–pathogen interactions. Thus, TcCRT is a potent virulence factor that inhibits the angiogenesis and a likely responsible, for at least in important part, of the antitumor effects of *T. cruzi* infection.

## In Eukaryotes, Calreticulin, an ER-Resident Chaperone Protein, Mediates Antitumor Properties

Calreticulin (CRT) is a 45 kDa protein, mainly residing in the ER (18). CRT participates in a variety of physiological and pathological processes in different cellular types (19). Thus, CRT contributes in multiple physiological processes such as control of glycoprotein folding quality system and binding to monoglucosylated high mannose glycans (20). Moreover, CRT is involved in quality control process during protein synthesis, including integrins, surface receptors, and transporters (21), and it is considered as an intracellular Ca^2+^ regulator (22).

Calreticulin is also found in the cytosol, nucleus, secretory granules, on the plasma membrane, and free in the extracellular milieu (18), accelerating cutaneous wound healing (23–25) and regulating cell adhesion by interacting with the cytosolic tail of the integrin alpha subunit (18); nuclear export of some steroid hormone receptors (26–28) and the stability or translation of a variety of RNAs (29–33). CRT is an mRNA binding protein that regulates mRNA stability (19).

Calreticulin also participates in the immune response against apoptotic cancer cells (34–38), and surface exposure of CRT participates as an “eat me” signal required for phagocytosis on dying tumor cells (39). Tumor tissues express significant higher levels of CRT compared to normal tissues (40). Indeed, its expression is related to the clinical stage and lymph node metastasis in several types of cancer (41, 42).

Over 40 functions have been described for human CRT (HuCRT) (43). These functions reside in three different domains: globular N-terminal (N), proline-rich (P), and acidic C-terminus (18). HuCRT and its N-terminal fragment bind laminin (44) with antiangiogenic properties *in vitro* and *in vivo* (45, 46) and inhibit the growth in several tumor models (47–49).

Vasostatin, a CRT 180 amino acid N-terminal fragment, is an endogenous inhibitor of angiogenesis and suppressor of tumor growth. It inhibits vascular endothelial growth factor (VEGF)-induced endothelial cell (EC) proliferation and tube formation in Matrigel and induces cell apoptosis under oxygen deprivation (50).

Calreticulin is present in humans (51), insects (52, 53), nematodes (54–57), protozoans (58–61), and plants (62). A high identity is shared among CRTs from different species. Thus, *Onchocerca volvulus, Schistosoma mansoni*, and *Leishmania donovani* share 50% of the identity in amino acid sequence with HuCRT.

Examples of important evasive strategies performed by CRTs from different parasite species are *Amblyomma americanum* [secretes CRT during the feeding process (63)] and *Schistosoma cercariae* [uses CRT in the penetration of gland cells or skin and parasite migration (54)].

## How Does *T. cruzi* Calreticulin Participate in the Host–Parasite Interplay?

Given the important pleiotropic HuCRT behavior, the CRT model opens interesting research opportunities on how this protein, alone or interacting with others, intervenes in the host–parasite interactions.

For 25 years now, our laboratory has worked with TcCRT. This protein is coded by only one gene with a variable number of copies whose involvement in TcCRT expression will depend on the *T. cruzi* clone and strain studied (unpublished data). A TcCRT gene was cloned, sequenced, and expressed in our laboratory in 1991 (58). We identified variable low plasma levels of anti-native TcCRT antibodies in *T. cruzi*-infected humans (64), thus revealing the immunogenic capacity of the native protein.

*Trypanosoma cruzi* calreticulin also binds monoglucosylated glycans (60) and participates in the maturation of cruzipain, a lysosomal protease (65) present in *T. cruzi*. Although TcCRT locates mainly in the ER, it is also found in the Golgi complex, reservosomes, flagellar pocket, cell surface, cytosol, nucleus, and kinetoplast (66, 67). However, the mechanisms involved in these diverse TcCRT localizations are unknown. Thus, TcCRT, in spite of its KEDL-ER retention sequence [KDEL in mammal CRTs (18)], translocates from the ER to the extracellular environment (Figures 1A,B) where, besides inhibiting complement (66) and acting as a virulence factor (68), it mediates antitumor effects.

In spite of the long evolutionary distance, TcCRT still shares 50% of overall sequence homology with HuCRT, reaching up to 80% in critical functional domains. Moreover, the general globular N-domain, responsible of antiangiogenic properties and the structural features of the extended arm P-domain also share structure homologies, thus announcing the possibility of functional similarities (69).

Two important TcCRT functions may explain the relationship between *T. cruzi* infection and cancer. First, TcCRT is an important complement inhibitor (Figure 1D) and virulence factor (Figure 1E). Second, TcCRT inhibits angiogenesis (Figure 1C). Both functions are central to inhibit tumor growth.

## TcCRT is an Important Virulence Factor in *T. Cruzi*

Similar to HuCRT (70, 71), TcCRT inhibits the complement system by interacting with C1 (Figure 1D), the first component of its classical pathway (66, 72–74). TcCRT is translocated from the ER to the area of flagellum emergence (Figure 1A) (66), where C1 is recruited by parasite-bound TcCRT and inhibited at the earliest complement activation step (C4b generation) (Figure 1D). TcCRT also affects the ability of C1s to activate C4, in a calcium-independent manner (74). Inhibition of C1 is a significant complement evasion strategy, with consequences in the host–parasite relationships. Although HuCRT and TcCRT prevent binding of the serine proteases to C1q, they do not displace the serine proteases from the preformed stabilized C1 (C1q, r_2_, and s_2_) complex (74). TcCRT also binds to MBL and Ficolins (75). C1, MBL, and Ficolins are three complement “danger signal” recognition macromolecular modules present in plasma. These molecular complexes are genetically, structurally, and functionally related, but they differ in the nature of the recognized danger signals (76). More recently, we have proposed that L-Ficolin binds TcCRT, inhibiting the lectin pathway. This inhibition may represent other *T. cruzi* strategy to inhibit the host immune response (75). In agreement with these findings, TcCRT is present on the parasite surface co-localizing with C1q (66).

Human CRT is also a membrane receptor for C1q [cC1qR (77)], and it may bridge TcCRT on the parasite surface with HuCRT present on the host cell (Figure 1E) (78). The TcCRT/C1q/HuCRT synapsis represents the culmination of an important molecular mimicry strategy. Apoptotic cells to be phagocytized use a similar mechanism (34, 36, 37). The CRT/C1q complex is recognized as an “eat me” signal by cC1qR on phagocytes. This signal is also used by *T. cruzi* as an “apoptotic mimicry” strategy (i.e., by capturing C1 in the area of flagellum emergence), thus facilitating the invasion/infectivity of host cells (79). This TcCRT-C1q-mediated parasite infectivity correlates with significant increases in TcCRT mRNA levels during early (cell contact and penetration) infection stages (36, 66, 68, 69, 72, 79). The TcCRT–C1q interaction can be prevented with anti-TcCRT F(ab′)_2_ fragments (devoid of the C1-binding Fc domains) (80). Indeed, passive immunization of mice with these fragments decreases infectivity (68). Congenital transmission is an important *T. cruzi* transmission pathway. Human pregnancy is a condition of elevated circulating CRT (81, 82). Moreover, human placenta expresses high CRT levels (83). We have recently proposed that the TcCRT/C1q/HuCRT interaction is very important in an *ex vivo* model of infection of human placenta (84), indicating a possible mechanism to explain the congenital transmission.

## TcCRT Participates in the Inhibition of Tumor Growth

Cancer is omnipresent in human history, and it also affects most of the living animal species, as a natural phenomenon of sporadic cellular dysfunction. Mammary, prostate, lung, cervix/uterine are just a few examples of cancer that, taken together, have epidemic proportions.

Interestingly, in patients infected with *T. cruzi*, cancer is rare (10, 12). About 80 years ago, Roskin, Ekzempliarskaia, and Klyuyeva, researchers from the former Soviet Union, postulated an experimental anticancer toxic activity derived from this infection. When they inoculated *T. cruzi* extracts, directly in a peritumoral area, in different tumors, both in experimental animals and in humans, similar results related to reduction of tumor size were obtained (10–13, 85, 86). More recently, the parasite capacity to infect preferentially tumor cells, as compared to normal host cells, was described (8). Although, in general, these data suggest an antagonism between *T. cruzi* infection and tumor growth (8), and research progress in these areas was seriously hampered by the intense international political problems of those years (i.e., the Cold War) (11). Although several publications on these issues have appeared during the last decades, the molecular basis of this phenomenon has remained elusive.

We propose that TcCRT is an important mediator of the antitumor effects of *T*. *cruzi* infection. Similar to HuCRT, TcCRT is antiangiogenic in *in vitro, ex vivo*, and *in vivo* models (Figure 1C) (3, 87, 88). Moreover, TcCRT inhibits the growth of a mammary adenocarcinoma and a melanoma in different experimental animal models (3, 87–89). The inhibition of tumor angiogenesis was proposed as a cancer therapy almost 40 years ago (90). For this reason, molecules or drugs with capacity to inhibit angiogenesis are currently applicable to a wide variety of tumors, often as a complement to other therapies (91).

*Trypanosoma cruzi* calreticulin and its N-terminal domain (N-TcCRT) were studied in different experimental set ups in mammals, *Homo sapiens* included (3). Thus, rTcCRT and its N-TcCRT inhibit capillary growth *ex vivo* in *Rattus rattus* aortic rings, morphogenesis, proliferation, and chemotaxis in human umbilical cord endothelial cells (HUVECs) (3) and *in vivo* angiogenesis in the *Gallus gallus* chorioallantoid membrane (CAM) assay (87). TcCRT was overall more effective, in molar terms, than HuCRT (3). Interestingly, in the CAM assay, the antiangiogenic TcCRT effect was fully reverted by polyclonal antibodies against rTcCRT (88).

In agreement with the previously described facts, the *in vivo* antitumor capacity of *T. cruzi* infection is paralleled by the inoculation of rTcCRT, with inhibits by 60–70% the time-course development of a murine mammary methotrexate multiresistant adenocarcinoma (TA3-MTX-R) (7).

## *T. Cruzi* Infects Neoplastic Cells and Promotes an Immune Response

Native TcCRT (nTcCRT) on the parasite contacts ECs, mediating internalization of *T. cruzi* and inhibition of tumor growth. This nTcCRT/EC contact may be indirect, mediated by C1q (Figure 1E) or by direct binding to scavenger receptors (SRs) (Figure 1F). TcCRT has affinity for collagenous structures, a possible explanation for its binding to human C1 and to SRs (66, 68). Fluid-phase Fucoidan, bearing extensive collagen-like sequences, inhibits the binding of CRT to SR-A present on both phagocytic cells (92) and the internalization of TcCRT by ECs (3).

## Is Native TcCRT Responsible for the Antitumor Effect of *T. Cruzi* Infection?

Recombinant TcCRT has important *in vivo* antiangiogenic and antitumor activities (3, 88). The antitumor effect of *T. cruzi* extract has been recently reproduced in a rat model. Experimental animals showed a strong cytotoxic response against tumor, with activation of CD4^+^ and CD8^+^ T cells and splenocytes. Moreover, a humoral adaptive immune response is generated. These anti-*T. cruzi* antibodies cross-reacted with tumor cells, inducing an antibody-dependent cellular toxicity *in vitro* (93). In a mouse model, we have reverted the antitumor effect of a *T. cruzi* epimastigote extract with specific antibodies against rTcCRT. Moreover, anti-rTcCRT F(ab′)_2_ antibodies (devoid of their capacity to interact with C1) neutralize the antitumor activity of *T. cruzi* infection, thus identifying nTcCRT as a mediator of this effect (unpublished data).

## How Does TcCRT Inhibit Tumor Growth in Individuals Infected with *T. Cruzi*?

We propose that, during *T. cruzi* infection, nTcCRT mediates key alterations in the tumor cell microenvironment leading to an adaptive immune response, with significant antitumor effects. Once in the circulation, *T. cruzi* must swiftly invade ECs (Figures 1E,F). Translocated-exteriorized TcCRT (Figures 1A,B) (92) will recruit and inactivate plasma complement C1 (Figure 1D) and inhibits angiogenesis (Figure 1C). This will allow the parasite to contact ECs *via* cC1qR (Figure 1E) (77, 94). Otherwise, the chaperone protein could interact directly with SR-A1 on ECs (Figure 1F) (95–97). Both pathways may lead to antiangiogenesis and generate a stressful environment where tumor cells will externalize their CRT, as previously shown with other stressing agents, such as Antracyclins (37). C1 recruitment and increased tumor cell phagocytosis by dendritic cells will follow (Figure 1H).

On the other hand, an adaptive immune response may be invoked by inoculated TcCRT or by its native counterpart timely externalized by infecting trypomastigotes (66) or present in epimastigote extracts (75). The chaperone protein should reach the surface of tumor cells (or ECs), thus generating a site for C1 binding (Figure 1G), followed by phagocytosis of these complexes by dendritic cells (Figure 1H). Targeting these activities on tumor cells should be favored by the parasite tropism for these tissues. The relevant novelty of parasite TcCRT is its difference in amino acidic sequence with the mammal (murine, in this case) counterpart. This difference may reach 50%, while mammal CRTs differ among them by no more than 10% (73). Upon arrival to the regional lymph nodes, these dendritic cells will present antigenic peptides derived from TcCRT, thus activating cytotoxic T lymphocytes, among other possibilities. Whether tumor cells can cross-present peptides derived from endocytosed TcCRT to cytotoxic T cells (Figure 1I) is a matter of current research in our laboratory. Activated cytotoxic T cells should then return to the tumor site and act against neoplastic tumor cells. Activation of CD4^+^ T cells *via* MHC II presentation, with stimulation of B cells and resulting ADCC against tumor cells, is a possibility that should also be entertained.

In our murine models, these antitumor effects are better performed by TcCRT, as compared to HuCRT. Among mammals, CRTs are at least 95% homologous in amino acidic differences. CRT immunogenicity across mammal species is thus restricted. On the other hand, because of extensive evolutionary distances, TcCRT amino acidic sequence differs by 50% with its mammal counterparts. Thus, TcCRT is more capable of generating immunogenic epitopes on the surface of mammal tumors. Recently, the expression of CRT has been correlated with a favorable prognosis of cancer. The high expression of CRT on tumor cells has been associated with a high density of infiltrating mature dendritic cells and effector memory T-cell subsets, suggesting that CRT triggers the activation of an adaptive immune response in the tumor microenvironment (98). Thus, TcCRT expressed and secreted by the parasite may be also important in this regard.

## Concluding Remarks

Infection with *T. cruzi* correlates with increased resistant to tumors. Since, during infection, nTcCRT is translocated to the parasite exterior and experimental parenteral administration of rTcCRT mimics the antitumor effects of the infection, nTcCRT is the most likely responsible molecule for these effects. Moreover, the antitumor effects of parasite infection can be specifically reverted by anti-rTcCRT antibodies. Since, in a large set of experimental animals treated with rTcCRT, no clinical deleterious effects have been detected by standard clinical veterinary criteria, we can now propose that rTcCRT or derived domains are interesting immunological tools to be considered in more advanced preclinical trials (e.g., rTcCRT capacity to bind to human mammary tumor cell lines *in vitro*, to subsequently incorporate C1, with increased capacity to induce phagocytosis).

## Author Contributions

GR-T, PA, and AF designed experiments. GR-T and PA performed experiments. GR-T, PA, and AF interpreted the data. GR-T, PA, and AF generated key reagents. GR-T, PA, and AF wrote, revised, and edited the manuscript. GR-T, PA, and AF approved the manuscript.

## Conflict of Interest Statement

The authors declare that the research was conducted in the absence of any commercial or financial relationships that could be construed as a potential conflict of interest.
